# Meta-Analysis of Tumor Stem-Like Breast Cancer Cells Using Gene Set and Network Analysis

**DOI:** 10.1371/journal.pone.0148818

**Published:** 2016-02-12

**Authors:** Won Jun Lee, Sang Cheol Kim, Jung-Ho Yoon, Sang Jun Yoon, Johan Lim, You-Sun Kim, Sung Won Kwon, Jeong Hill Park

**Affiliations:** 1 College of Pharmacy and Research Institute of Pharmaceutical Sciences, Seoul National University, Seoul, 08826, Republic of Korea; 2 Department of Biomedical Informatics, Center for Genome Science, National Institute of Health, KCDC, Choongchung-Buk-do, 28159, Republic of Korea; 3 Department of Biochemistry and Department of Biomedical Sciences, Ajou University School of Medicine, Suwon, 16499, Republic of Korea; 4 Department of Statistics, Seoul National University, Seoul, 08826, Republic of Korea; University of Nebraska Medical Center, UNITED STATES

## Abstract

Generally, cancer stem cells have epithelial-to-mesenchymal-transition characteristics and other aggressive properties that cause metastasis. However, there have been no confident markers for the identification of cancer stem cells and comparative methods examining adherent and sphere cells are widely used to investigate mechanism underlying cancer stem cells, because sphere cells have been known to maintain cancer stem cell characteristics. In this study, we conducted a meta-analysis that combined gene expression profiles from several studies that utilized tumorsphere technology to investigate tumor stem-like breast cancer cells. We used our own gene expression profiles along with the three different gene expression profiles from the Gene Expression Omnibus, which we combined using the ComBat method, and obtained significant gene sets using the gene set analysis of our datasets and the combined dataset. This experiment focused on four gene sets such as cytokine-cytokine receptor interaction that demonstrated significance in both datasets. Our observations demonstrated that among the genes of four significant gene sets, six genes were consistently up-regulated and satisfied the p-value of < 0.05, and our network analysis showed high connectivity in five genes. From these results, we established CXCR4, CXCL1 and HMGCS1, the intersecting genes of the datasets with high connectivity and p-value of < 0.05, as significant genes in the identification of cancer stem cells. Additional experiment using quantitative reverse transcription-polymerase chain reaction showed significant up-regulation in MCF-7 derived sphere cells and confirmed the importance of these three genes. Taken together, using meta-analysis that combines gene set and network analysis, we suggested CXCR4, CXCL1 and HMGCS1 as candidates involved in tumor stem-like breast cancer cells. Distinct from other meta-analysis, by using gene set analysis, we selected possible markers which can explain the biological mechanisms and suggested network analysis as an additional criterion for selecting candidates.

## Introduction

Cancer stem cells (CSCs) have been known to cause rapid tumor formation and recurrence in cancer cell populations [1]. In various solid tumors, including breast, brain, pancreatic cancer and ovarian cancers, CSCs were observed to be highly resistant cells to chemotherapy. Additionally, CSCs appear to be more aggressive and have been known to exhibit epithelial-to-mesenchymal-transition (EMT) characteristics [2]. Thus, the investigation of CSCs is important for cancer research [3]. Because sphere cells are known to maintain the properties of CSCs, the method of comparing sphere cells with adherent cells is widely accepted for investigating mechanisms underlying CSCs [2]. Several studies have identified CD24-/CD44+, aldehyde dehydrogenase activity (ALDH1) and ABC transporter dependent Hoechst side population (SP) as tumor initiating cells-related markers but these markers showed no correlation with CSCs [1, 2]. Therefore, the identification of CSC-related markers remains a challenging issue in cancer therapy [1, 2].

To increase the statistical power, meta-analysis integrates results from related studies and provides reliable and general results, and this method is inexpensive because we can perform combined meta-analysis on available microarray datasets from open sources such as Gene Expression Omnibus (GEO) [4, 5]. In this study, we combined different gene expression profiles from several studies that investigated tumor stem-like breast cancer cells, and each gene expression profile consisted of sphere cells and adherent cells [2, 3, 6]. To conduct a meta-analysis, we obtained three gene expression profiles that used Affymetrix Gene Chip Arrays from GEO and combined these datasets into one using the ComBat method [7]. We also generated sphere cells derived from the adherent breast cancer cell line MCF-7 and acquired our gene expression data using Illumina Gene Chip Arrays.

So far, meta-analysis have suggested four categories of techniques including vote counting, combining ranks, combining p-values and combining effect sizes [5, 8]. However, these methods did not consider the information of biological process but only statistical process. In our meta-analysis, we compared gene expression differences between sphere and adherent cells using gene set analysis of datasets generated with the Affymetrix and Illumina platforms. The approach of identifying individual genes with statistical significance is not sufficient for interpreting biological processes from gene expression profiles [9]; thus, the analysis of gene sets, i.e., the concepts of multiple functionally related genes, could provide a robust approach for translating the biological significance of gene expression profiles [10, 11]. Previous studies have demonstrated the successful application of gene set analysis using gene expression data [12–14]. Using a cut-off of *p* < 0.001, we determined several significant gene sets using Affymetrix and Illumina datasets and found four significant gene sets that were significant in both platforms. For validation, we used leave-one-out cross-validation in each platform and calculated the accuracy of the significant gene sets using prediction analysis for microarrays (PAM) and also evaluated the classification performance of significant gene sets using Kernel-based Orthogonal Projections to Latent Structures (K-OPLS) [15]. From the four significant gene sets, we selected individual gene based on p-values and expression directions using the Globaltest R package [9, 16, 17]. Distinct from other meta-analysis, we selected individual markers which can explain the mechanisms underlying tumor stem-like breast cancer cells by applying gene set analysis to meta-analysis.

Furthermore, to consider the network properties of the candidates, we determined their connectivity, the statistical value for evaluating the degree of correlation with other genes using weighted correlation network analysis (WGCNA) [18]. In the network analysis, a hub gene is highly connected to other genes and considered to be central to the network architecture [19]. Some biological studies have reported the importance of hub genes and revealed the importance of intramodular hub genes [19]. For example, yeast survival was found to be associated with highly connected hub genes, and several studies demonstrated that hub genes are preserved across species [19].

From the gene set and network analysis, we considered both significance and connectivity for detecting the candidates which involved in tumor stem-like breast cancer cells. Furthermore, we validated the candidates using quantitative reverse transcription-polymerase chain reaction (RT-PCR). Our results demonstrate that the concept of meta-analysis integrated with gene set and network analysis may be useful for investigating the mechanisms underlying tumor stem-like breast cancer cells.

## Materials and Methods

### Data collection

**Fig 1:**
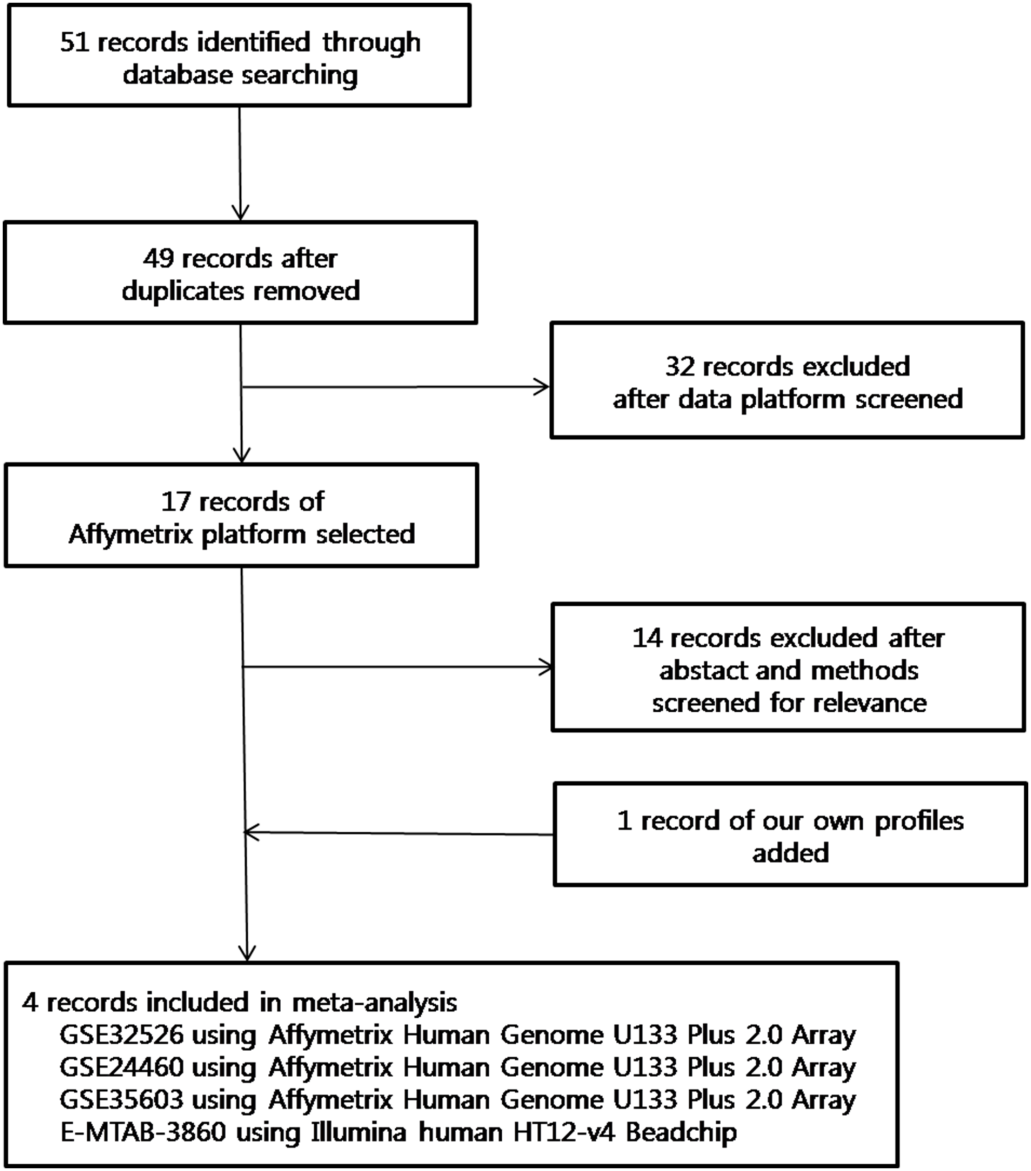
Flow diagram of database searching and the process for selecting studies.

Fig 1 shows the process of database searching and study selection. For data collection, we searched two databases, Gene Expression Omnibus (GEO, www.ncbi.nlm.nih.gov/geo/) and ArrayExpress (www.ebi.ac.uk/arrayexpress) and used the search terms including “cancer stem”, “breast”, “sphere”, “mammosphere” and “tumor stem-like”. With these search terms, we found 51 studies and removed two duplicates. Among these 49 studies, we selected 17 studies using Affymetrix Human Genome U133 Plus 2.0 Array for expression profiling to preprocess expression data of the same platform. From the 17 selected studies, final three studies were selected and these studies included following features: (1) the study provided adequate expression data conducted in human breast cancer tissue and (2) the study included expression data of sphere and adherent cells for investigating tumor stem-like breast cancer cells. In addition to Affymetrix, we obtained gene expression profile using Illumina human HT12-v4 Beadchip. In sum, meta-analysis was performed by using three datasets from Affymetrix platform and one dataset from Illumina platform.

### Cell culture and gene expression profiling

The MCF-7 breast cancer cell line was obtained from American Type Culture Collection (ATCC, Manassas, VA) and maintained in DMEM medium supplemented with 10% fetal bovine serum. Single cell suspensions of MCF-7 cells were seeded at a density of 5 x 10^5^ cells/mL in DMEM/F12 containing 1 x B27 supplement (Life Technologies, Carlsbad, CA), 20 ng/mL basic fibroblast growth factor (R&D Systems, Minneapolis, MN), 20 ng/mL recombinant epidermal growth factor (Life Technologies, Carlsbad, CA), 100 U/mL penicillin, and 100 μg/mL streptomycin, and they were seeded in an ultralow adherence dish (Corning, Corning, NY). Cultures were fed twice a week and sub-cultured by weekly trypsinization and dissociation with a 23-gauge needle. Single cells were pelleted and suspended in mammosphere media at 5 x 10^5^ cells/mL in ultralow adherence dishes [20]. Total RNA was extracted from tumor specimens using the mirVana^™^ RNA isolation kit (Ambion, Inc., Carlsbad, CA) according to the manufacturer’s instructions. Total RNA (500 ng per sample) was used for cRNA production using the Illumina TotalPrep RNA amplification kit (Ambion, Inc., Carlsbad, CA). The integrity and quantity of the total RNA were assessed with a NanoDrop (Thermo Scientific, Wilmington, DE) and Bioanalyzer (Agilent Technologies, Santa Clara, CA). cRNA was used for hybridization to the Illumina human HT12-v4 Beadchip gene expression array (Illumina) according to the manufacturer's protocol. The hybridized arrays were scanned, and fluorescence signals were obtained using the Illumina Bead Array Reader (Illumina, San Diego, CA).

### Preprocessing

The Affymetrix Human Genome U133 Plus 2.0 Array was used for gene expression profiling for three datasets including GSE32526, GSE24460 and GSE35603. To normalize the gene expression of these three datasets, we used robust multi-array analysis (RMA) in the affy R package [21]. After normalization, we removed severe batch effects that were found in the three different datasets using the ComBat method is the sva R package so that we could integrate the three datasets into one (S1 Fig). To directly model the batch effects, the sva package uses the Combat method function [7]. In high-throughput biological experiments, there are potentially a large number of environmental and biological variables that are unmeasured and may have a large impact on measurements. In cases such as these, the Combat method is appropriate for removing these artifacts. To reduce dependence, the stabilizing error rate estimates and improves reproducibility, and the Combat method removes batch effects and uses surrogate variables in differential expression analyses [22–24]. Using an empirical Bayesian framework, that Combat method can be used for high-dimensional data matrices, and the output is a corrected expression profile [7]. For preprocessing the gene expression profiles using the Illumina platform, the signals were log2 transformed and normalized by quantile normalization. Then, we converted the gene labels into Entrez IDs using Database for Annotation, Visualization, and Integrated Discovery (DAVID) software [25].

### Gene set analysis

For gene set analysis, we used the “gage” R package. The “gage” R package uses the Generally Applicable Gene-set Enrichment (GAGE) method. The previously used gene set analysis methods such as GSEA and PAGE have some limitations in handling datasets of different sample sizes or experimental design. GAGE expands the applicability of gene set analysis by addressing these limitations. Additionally, GAGE consistently demonstrates better results when compared with previous gene set analysis methods in three major aspects: (a) consistency across repeated studies/experiments, (b) sensitivity and specificity, and (c) biological relevance of the regulatory mechanisms inferred. From both published and unpublished microarray studies, GAGE has revealed novel and relevant regulatory mechanisms [26].

To select significant gene sets in sphere and adherent cells, we applied the “gage” R package to each gene profile generated by the Affymetrix and Illumina platforms. Gene sets derived from KEGG were evaluated by their p-values for differences between treatments and controls. We calculated the p-value of each gene set for differences between sphere and adherent cells using “gage” and then selected significant gene sets with a cut-off of *p* < 0.001. From the Affymetrix and Illumina platforms, we obtained several significant gene sets. We then selected four gene sets that satisfied *p* < 0.001 in both platforms. Additionally, the “gage” R package calculated q-values as a false discovery rate (FDR) based on an adjustment of the p-value using the Benjamini and Hochberg procedure [26].

### Validation and selecting candidates

To validate the four significant gene sets, we used leave-one-out cross validation. In each platform, one of the total samples was removed, a prediction model was developed using the remain samples, and the left out sample was then predicted for sphere or adherent cells [27].

Leave-one-out cross-validation was conducted by using prediction analysis for microarrays (PAM) to develop a prediction model and classification. Using the nearest shrunken centroid method, PAM classifies samples from gene expression data [28]. Samples were classified by the subsets of genes that characterized each class. Several studies have used PAM to predict classes of gene expression data [29–32]. After conducting validation, the accuracy of each significant gene set was calculated for the two platforms.

Principle component analysis (PCA) was performed using the “princomp” function of Matlab, and we examined a 3D PCA plot for the expression values of each of the four gene sets and using PCA, Affymetrix and Illumina datasets each distributed 15 and 4 samples.

To determine the gene candidates, we obtained the p-values of genes in the four selected gene sets using the Globaltest R package in which p-values may be represented for each gene using the component test. By using p-values for the direction of expression, Globaltest evaluates each gene as a positive or negative association [16, 17]. A positive association indicates that the expression of a gene is up-regulated by treatment. In contrast, a negative association indicates that the expression of a gene is down-regulated by treatment. In our study, compared with adherent cells, a positive association indicates that the expression of a gene is up-regulated in sphere cells, and a negative association indicates that the expression of a gene is down-regulated in sphere cells. We selected gene candidates that satisfied a *p* < 0.05 in the same direction in both platforms.

To visualize the significance of the genes in the significant gene sets, we generated a gene plot using the Globaltest R package. p-values of genes were set as bars, and the bars were colored in shades of red or green. Based on the comparison of sphere cells with adherent cells, the red bars indicated genes up-regulated in sphere cells, and the green bars indicated down-regulated genes in sphere cells.

For further understanding, we calculated the average fold-change of individual genes between adherent and sphere cells in both platforms and mapped the fold-changes in the KEGG pathway using the pathview R package (http://bioconductor.org/packages/2.12/bioc/html/pathview.html), which is a tool set for data integration and the visualization of pathways. Using pathview, a wide variety of biological data were mapped to the target pathways specified.

For additional validation, we evaluated the classification performance of the four significant gene sets, we used K-OPLS [15]. By allowing detection of unanticipated systemic variation such as instrumental drift, batch variability or unexpected biological variation, K-OPLS features enhanced interpretational capabilities and were well suited for the analysis of various biological data [15, 33]. We implemented 100-permutations and obtained the area under the curve (AUC) by using the K-OPLS R package. Based on the results of K-OPLS, we generated ROC curves.

### Network analysis

To determine the network properties of candidate genes, we applied network analysis to each significant gene set obtained by gene set analysis. We also calculated the connectivity of each gene involved in the selected gene sets using WGCNA to determine the hub genes. The WGCNA package implements an R package for weighted correlation network analyses e.g., co-expression network analysis using gene expression data [18]. In complex diseases, recent studies have demonstrated successful applications including the interaction between genotype data and co-expression modules [34–39]. WGCNA can be used to reduce microarray data from thousands of genes into clusters (modules) of highly correlated genes and to determine intramodular hub genes that are highly correlated with other genes [18]. The R package and its source code including additional material are freely available for download at http://www.genetics.ucla.edu/labs/horvath/CoexpressionNetwork/Rpackages/WGCNA.

### Reverse transcription-PCR

RT-PCR was performed to confirm the expression of candidate genes and a total of 1 μg RNA from each sample was used as a template for cDNA synthesis using a reverse transcriptase kit (Promega). An equal amount of cDNA generated with Taq DNA polymerase (Promega) was used in the PCR. S1 Table shows the list of final candidates and reference genes, including SNAI and ACTIN, and their sense and anti-sense primers for PCR amplification. PCR amplification was performed at an optimized annealing temperature, and the number of PCR cycles was 27 or 30 (S1 Table).

## Results

### Characteristics of Datasets

We used three Affymetrix Gene Chip Array gene expression profiles including GSE32526, GSE24460 and GSE35603, which all the research articles of these expression profiles from GEO were published between 2010 and 2012 (Table 1). GSE32526 is a gene expression profile dataset from human breast cancer patients, including 55-year-old and 85-year-old females that were divided into the highly tumorigenic S2N and weakly tumorigenic S2 categories [2]. We used the highly tumorigenic S2N data that had three replicates for sphere cells and three replicates for adherent cells derived from sphere cells. These were obtained by surgical treatment in accordance with the ethical standards of the responsible institutional committee at the University of Palermo on human experimentation [2]. From the GSE24460 dataset, we used parental MCF-7 and MCF-7/ADR cells [3]. Parental MCF-7 cells were wild-type and estrogen receptor-positive luminal subtypes [3, 40]. MCF-7/ADR cells were highly invasive sphere cells and cultured in high-dose doxorubicin every other passage, as described previously [3]. Parental MCF-7 cells had two replicates and MCF-7/ADR cells had two replicates [3]. From the GSE35603 dataset, we used three replicates from parental MCF-7 cells and two replicates from tumor stem-like cells derived from parental MCF-7 cells [6]. These parental MCF-7 cells were wild-type and estrogen receptor-positive [41]. We also added the gene expression profiles of Illumina platform, which had two replicates for parental MCF-7 and their mammosphere cells, respectively (Table 1). Our parental MCF-7 cells were luminal A subtypes and estrogen receptor-positive. Gene expression data are publicly available at ArrayExpress (www.ebi.ac.uk/arrayexpress) and the accession number is E-MTAB-3860.

**Table 1:** Datasets used for meta-analysis and their characteristics.

Datasets	Platforms	Adherent cells	Sphere cells
GSE32526	Affymetrix Human Genome U133 Plus 2.0 Array	3	3
GSE24460	Affymetrix Human Genome U133 Plus 2.0 Array	2	2
GSE35603	Affymetrix Human Genome U133 Plus 2.0 Array	3	2
E-MTAB-3860	Illumina human HT12-v4 Beadchip	2	2

### Gene set analysis and validation

**Fig 2:**
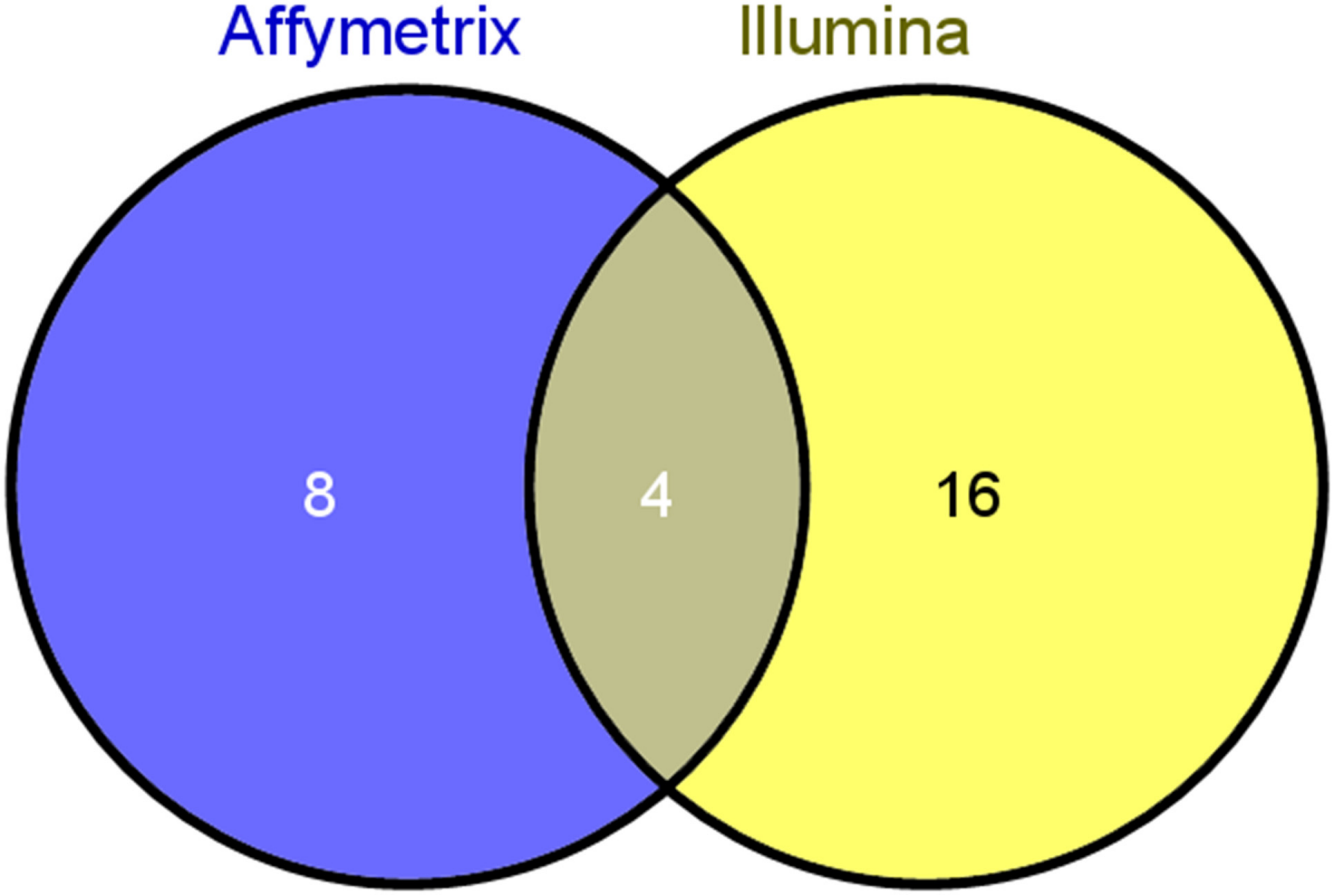
Venn diagram showing four gene sets derived from gene set analysis that satisfied *p* < 0.001 in the Affymetrix and Illumina platforms.

Using gene set analysis with a cut-off of *p* < 0.001, we obtained 12 and 20 significant gene sets each from the Affymetrix and Illumina platforms (S2 Table). We generated a Venn diagram using these significant gene sets to determine the commonly expressed gene sets (Fig 2). From the Venn diagram, we selected four gene sets, including cytokine-cytokine receptor interaction, valine, leucine and isoleucine degradation, systemic lupus erythematosus and DNA replication, which were common in both platforms. These four gene sets also satisfied a false discovery rate (FDR) < 0.05 in both platforms (Table 2, S2 Table). DNA replication demonstrated the highest significance for sphere and adherent cells (Table 2).

**Table 2:** Four gene sets that satisfied *p* < 0.001 in both the Affymetrix and Illumina datasets and their p-values, FDR, accuracy and AUC. P-values and FDR were calculated using the “gage” R package, and the accuracy was obtained from leave-one-out cross validation. AUC generated by K-OPLS for scoring classifiers of four gene sets.

Gene Sets	Affymetrix				Illumina		
	p-value	FDR	accuracy (%)	AUC	p-value	FDR	accuracy (%)
DNA replication	9.81E-05	0.008	87	0.939	6.62E-12	1.17E-09	100
Valine, leucine and isoleucine degradation	0.000729	0.016	73	0.816	0.000251	0.007	100
Cytokine-cytokine receptor interaction	0.000852	0.041	87	0.841	0.000575	0.010	100
Systemic lupus erythematosus	0.000978	0.041	93	0.982	0.000553	0.010	100

We then used leave-one-out cross validation to obtain the accuracy [42]. For each leave-one-out cross validation result, the output of positive or negative indicated that left out samples were classified as a sphere cell or adherent cell, respectively.

According to the concept of accuracy, a true positive (TP) indicates the number of sphere cells that were predicted to be sphere cells, and false positive (FP) indicates the number of adherent cells predicted to be sphere cells. In the same manner, a true negative (TN) indicated the number of adherent cells that were predicted to be adherent cells and a false negative (FN) was the number of sphere cells that were predicted to be adherent cells. From the TP, FP, TN and FN data, we calculated the accuracy of each significant gene set.

Table 3 lists the results of the leave-one-out cross validation. All of the Illumina samples were classified as TP or TN. For the Affymetrix samples, several were classified as FP or FN. In the cytokine-cytokine receptor interaction gene set, sample numbers 4 and 15 were classified as FP and FN, respectively. In the valine, leucine and isoleucine degradation gene set, sample numbers 7 and 8 were classified as FP, and sample numbers 10 and 15 were classified as FN. In the systemic lupus erythematosus gene set, sample number 15 was classified as FN. In the DNA replication gene set, sample numbers 4 and 10 were classified as FP and FN, respectively. Based on the results of the leave-one-out cross validation, we calculated the accuracy of the four significant gene sets. Table 2 demonstrates that these four significant gene sets had > 70% accuracy in the Affymetrix platform and 100% accuracy in the Illumina platform.

**Table 3:** Leave-one-out cross validation where one of the total samples was removed, a prediction model was developed using the remaining samples, and the left-out sample was then predicted for sphere or adherent cells in each platform. An output of 1 indicates that a left-out sample from adherent or sphere cells was predicted to be an adherent or sphere cell, respectively. An output of 0 indicates that a left-out sample from adherent or sphere cells was predicted as a sphere or adherent cell, respectively.

Gene Sets	Samples of Affymetrix															Samples of Illumina			
	Adherent cells								Sphere cells							Adherent cells		Sphere cells	
	1	2	3	4	5	6	7	8	9	10	11	12	13	14	15	1	2	3	4
Cytokine-cytokine receptor interaction	1	1	1	0	1	1	1	1	1	1	1	1	1	1	0	1	1	1	1
Valine, leucine and isoleucine degradation	1	1	1	1	1	1	0	0	1	0	1	1	1	1	0	1	1	1	1
Systemic lupus erythematosus	1	1	1	1	1	1	1	1	1	1	1	1	1	1	0	1	1	1	1
DNA replication	1	1	1	0	1	1	1	1	1	0	1	1	1	1	1	1	1	1	1

**Fig 3:**
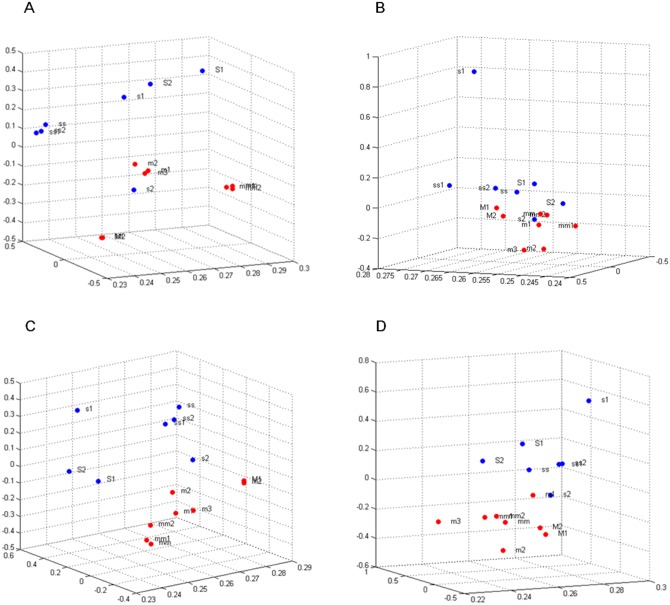
PCA plot in which m and s indicate the adherent and sphere cell samples in the GSE35603 dataset, M and S indicate adherent and sphere cell samples, respectively, in the GSE24460 dataset, and mm and ss indicate adherent and sphere cell samples, respectively, in the GSE32526 dataset. All 15 samples from the Affymetrix datasets were distributed by the expression of the four significant gene sets including **A**. Cytokine-cytokine receptor interaction **B**. Valine, leucine and isoleucine degradation **C**. Systemic lupus erythematosus **D**. DNA replication.

In the PCA plot, fifteen Affymetrix samples and four Illumina samples were distributed based on significance of the four gene sets (Fig 3, S2 Fig). Among these gene sets, the valine, leucine and isoleucine degradation gene set demonstrated poor classification, and this result was consistent with its lowest accuracy of 73% in the leave-one-out cross validation. In addition to PCA, we used K-OPLS and revealed > 0.8 AUC of the four significant gene sets in Affymetrix datasets. The receiver operating characteristic (ROC) curve of each four significant gene set was obtained using the results of K-OPLS and because Illumina datasets had small number of samples, K-OPLS method was not implemented in Illumina datasets (S3 Fig).

### Selecting candidate genes

Among the significant gene sets, we selected candidate genes as the possible marker. We considered the p-value and expression direction of individual gene using Globaltest to select the candidates.

Table 4 shows genes in significant gene sets that satisfied a *p* < 0.05 in both the Affymetrix and Illumina platforms. In the cytokine-cytokine receptor interaction gene set, IL12RB2, CXCL1 and CXCR4 were up-regulated in both platforms, but CXCL10, CXCL6 and TNFRSF11B were down-regulated. In the valine, leucine and isoleucine degradation gene set, ACADM, BCKDHB and HMGCS1 were up-regulated in both platforms, but PCCB and AOX1 down-regulated. In the systemic lupus erythematosus gene set, only HLA-DMA was up-regulated in both platforms. In the DNA replication gene set, no gene demonstrated a consistent direction of expression between the two platforms. Among these gene sets, the cytokine-cytokine receptor interaction and valine, leucine and isoleucine degradation gene sets contained many genes that demonstrated a consistent direction of expression in both platforms.

**Table 4:** P-values, direction and connectivity of genes that consist of significant gene sets and satisfied *p* < 0.05 in the Affymetrix and Illumina platforms. P-values and direction were obtained using the “Globaltest” R package. An up or down direction indicates that the expression of a gene is up- or down-regulated in sphere cells, respectively. Connectivity, scaled connectivity and clustering coefficients were obtained from WGCNA for each gene set.

Gene Sets	Genes	Affymetrix					Illumina				
		p-value	Direction	Connectivity	Scaled Connectivity	Clustering Coefficient	p-value	direction	Connectivity	Scaled Connectivity	Clustering Coefficient
Cytokine-cytokine receptor interaction	CXCL10	0.004	Down	7.339	0.484	0.217	0.038	up	58.222	0.932	0.460
	CXCL6	0.009	Down	4.709	0.311	0.139	0.028	up	57.983	0.928	0.459
	IL12RB2	0.013	Up	1.703	0.112	0.053	0.030	up	57.588	0.922	0.475
	CXCL1	0.014	up	2.619	0.173	0.082	0.011	up	60.767	0.972	0.475
	TNFRSF11B	0.022	down	1.641	0.108	0.040	0.009	up	61.191	0.979	0.478
	CXCR4	0.029	up	1.486	0.098	0.108	0.001	up	62.369	0.998	0.483
Valine, leucine and isoleucine degradation	PCCB	0.000	up	0.156	0.023	0.088	0.025	down	13.050	0.630	0.531
	ACADM	0.004	up	1.076	0.162	0.091	0.013	up	14.366	0.693	0.519
	BCKDHB	0.015	up	3.931	0.591	0.187	0.037	up	16.723	0.807	0.560
	AOX1	0.036	down	3.608	0.543	0.251	0.023	up	14.370	0.694	0.551
	HMGCS1	0.038	up	6.083	0.915	0.261	0.005	up	16.723	0.807	0.495
Systemic lupus erythematosus	HIST1H2BD	0.000	down	6.428	1.000	0.297	0.037	up	34.910	0.862	0.491
	HLA-DMA	0.001	up	1.531	0.238	0.209	0.049	up	34.737	0.858	0.501
	HIST2H3A	0.002	down	6.424	0.999	0.332	0.004	up	16.371	0.404	0.376
	HIST1H2BC	0.006	down	5.389	0.838	0.329	0.018	up	38.786	0.958	0.523
	H2AFJ	0.013	down	5.692	0.886	0.364	0.008	up	40.420	0.999	0.533
	SSB	0.026	up	1.982	0.308	0.092	0.025	down	37.591	0.929	0.514
	HIST2H2BE	0.045	down	6.190	0.963	0.314	0.000	up	39.830	0.984	0.529
DNA replication	RfC4	0.000	up	2.324	0.435	0.142	0.030	down	19.755	0.917	0.603
	RPA1	0.002	up	4.078	0.763	0.207	0.001	down	21.553	1.000	0.626
	MCM4	0.005	up	2.725	0.510	0.274	0.001	down	21.239	0.985	0.639
	MCM5	0.007	up	2.773	0.519	0.301	0.014	down	19.233	0.892	0.643
	MCM2	0.009	up	3.880	0.726	0.201	0.009	down	19.912	0.924	0.638
	FEN1	0.022	up	3.304	0.618	0.209	0.039	down	18.427	0.855	0.650
	RFC5	0.029	up	5.344	1.000	0.184	0.031	down	19.289	0.895	0.640

**Fig 4:**
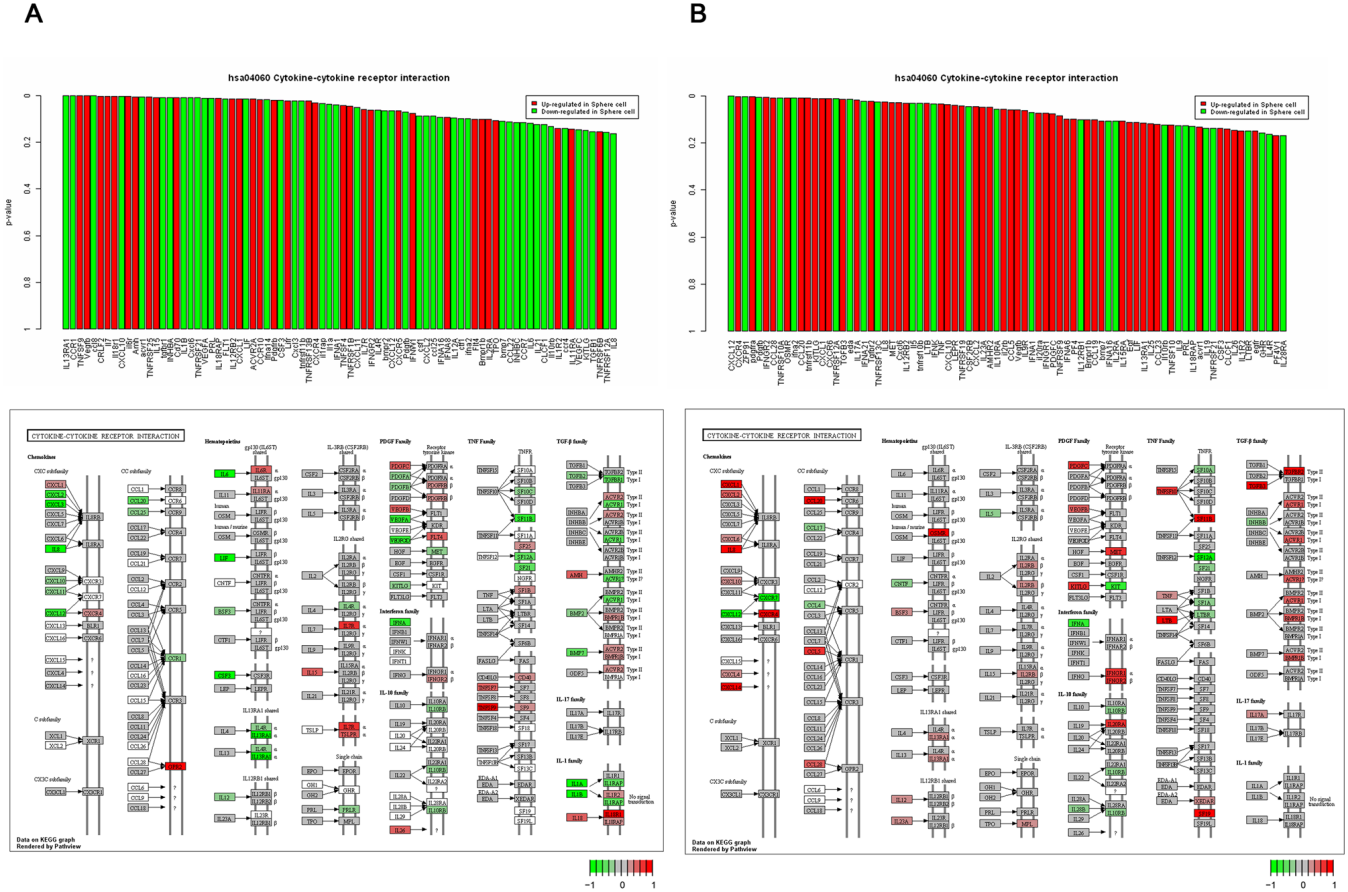
The top shows gene plots of the cytokine-cytokine receptor interaction gene set obtained from Globaltest. Red and green bars indicate genes that were up-regulated or down-regulated, respectively, in sphere cells. The bottom shows KEGG pathways including the fold-change of individual genes in the cytokine-cytokine receptor interaction gene set. **A**. Affymetrix datasets. **B**. Illumina datasets.

Fig 4 and S4 Fig show gene plots of the cytokine-cytokine receptor interaction and valine, leucine and isoleucine degradation gene sets from the Affymetrix and Illumina datasets. For the cytokine-cytokine receptor interaction gene set, the Affymetrix datasets show that there are 21 genes including TNFSF9, VEGFB, CRLF2, IL7 and IL18R1 that were significantly up-regulated, and 22 genes, including IL13RA1, CCR1, CCL8, CXCL10 and ACVR1, which were significantly down-regulated in sphere cells (Fig 4A). In the Illumina datasets, 25 genes, including CXCR4, PDGFRA, IFNGR2, CCL28 and OSMR, were significantly up-regulated, and 13 genes, including CXCL12, ZFP91, PDFGB, TNFRSF10A and IFNA2, were significantly down-regulated in sphere cells (Fig 4B). The Affymetrix datasets showed that 7 genes in the valine, leucine and isoleucine degradation gene set, including PCCB, HADH, ALDH9A1, ACADM and ALDH7A1, were significantly up-regulated, and only AOX1 was significantly down-regulated in sphere cells (S4 Fig). A total of 10 genes, including HMGCS1, AUH, ABAT, ACADM and AOX1, were significantly up-regulated, and only PCCB was significantly down-regulated in sphere cells in the Illumina datasets (S4 Fig). The Illumina datasets demonstrated higher expression than the Affymetrix datasets for the cytokine-cytokine receptor interaction and valine, leucine and isoleucine degradation gene sets.

The bottom portion of Fig 4 illustrates that the Illumina datasets demonstrate a greater number of activated chemokine-related genes than the Affymetrix datasets. For the chemokine-related genes, CXCL1 and CXCR4 in the Illumina datasets demonstrated higher up-regulation than that in the Affymetrix datasets. Additionally, of the TNF and TGF-β family-related genes, Illumina datasets demonstrate greater up-regulation. Finally, we selected IL12RB2, CXCL1, CXCR4, ACADM, BCKDHB and HMGCS1 from the cytokine-cytokine receptor interaction and valine, leucine and isoleucine degradation gene sets as candidate genes that were up-regulated in both platforms.

### Network analysis

To consider the network properties of the candidates, we performed network analysis using each of the four significant gene sets. WGCNA results (Table 4) show the network statistics including the connectivity of genes in the selected gene sets that satisfied *p* < 0.05 in both platforms and the connectivity has been associated with important properties of proteins and metabolic networks and indicates the sum of correlation strengths between a target gene and all of its neighbors [43, 44]. Scaled connectivity is scaled by the highest connectivity in each gene set i.e., connectivity/max (connectivity), and it is used to compute the hub gene significance [19, 45]. To search for hub genes, we evaluated the target genes with scaled connectivity. The clustering coefficient of a target gene is a density measurement of the local connections or relatedness of each gene [46, 47].

Among the genes in the cytokine-cytokine receptor interaction gene set, CXCL10 demonstrated the highest scaled connectivity at 0.484 in Affymetrix datasets but had the opposite expression in both platforms. In the Illumina datasets, CXCR4 had the highest scaled connectivity at 0.998 and up-regulation in both platforms. In the valine, leucine and isoleucine degradation gene set, HMGCS1 demonstrated the highest scaled connectivity at 0.915 and 0.807 in the Affymetrix and Illumina datasets, respectively. In addition, HMGCS1 was up-regulated in both platforms. Among the genes in the systemic lupus erythematosus gene set, HIST1H2BD demonstrated the highest scaled connectivity at 1.000 in the Affymetrix datasets. However, HIST1H2BD demonstrated the opposite expression between two platforms. In the Illumina datasets, H2AFJ demonstrated the highest scaled connectivity at 0.999 but had the opposite expression between the platforms. In the DNA replication gene set, RFC5 and RPA1 demonstrated the highest scaled connectivity in the Affymetrix and Illumina datasets, respectively, but they had the opposite expression between datasets.

CXCR4 had the highest clustering coefficient in the cytokine-cytokine receptor interaction gene set in the Illumina dataset. In the valine, leucine and isoleucine degradation gene set in the Affymetrix datasets, HMGCS1 had the highest clustering coefficient.

Among the significant genes of cytokine-cytokine receptor interaction and valine, leucine and isoleucine degradation gene sets, those of Illumina datasets demonstrated higher connectivity and scaled connectivity than those of Affymetrix datasets. Importantly, all significant genes of cytokine-cytokine receptor interaction gene set demonstrated > 0.9 scaled connectivity in Illumina datasets.

### Reverse transcription-PCR

**Fig 5:**
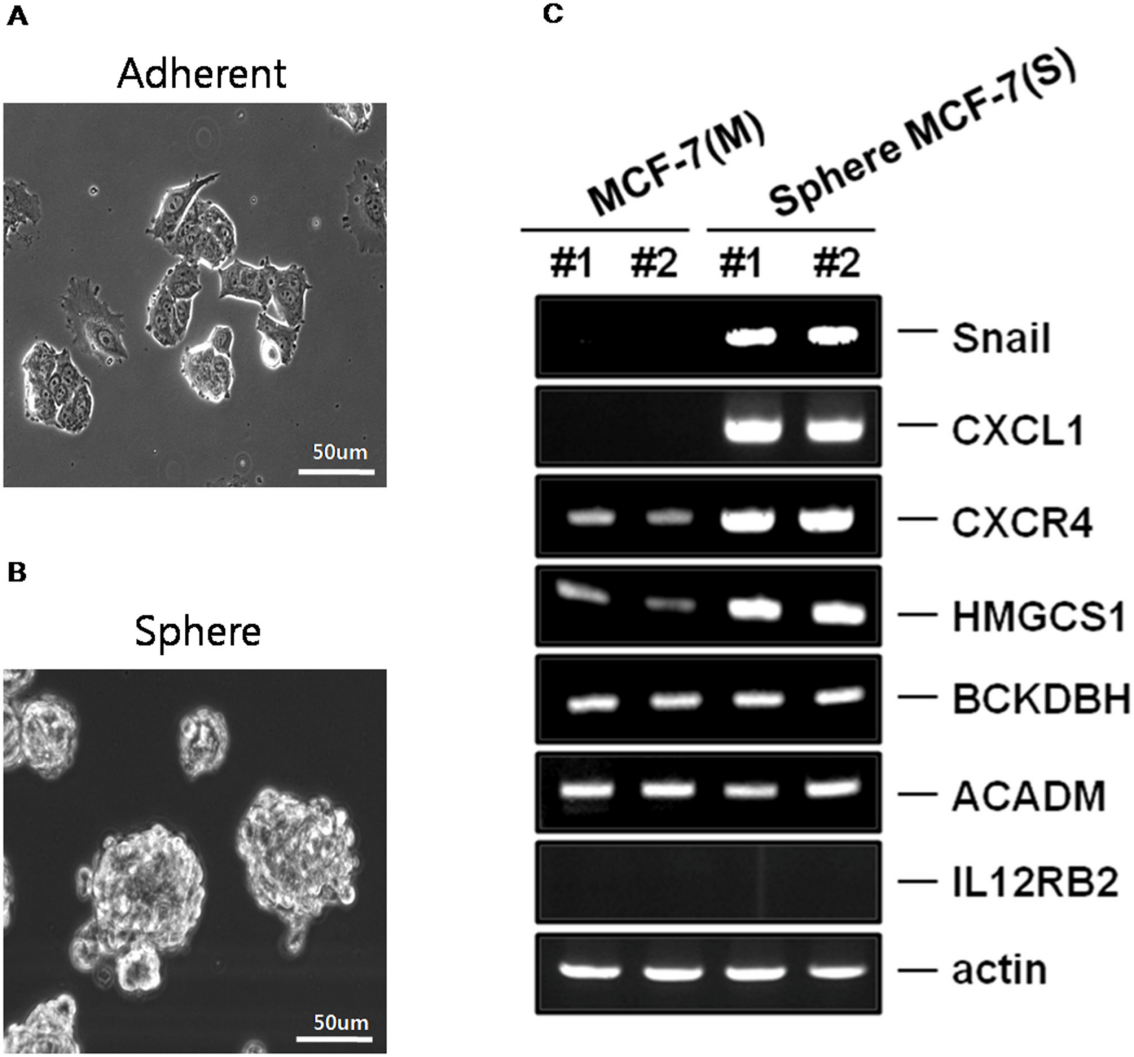
Microscopic images of the MCF-7 and MCF-7-derived sphere cells and quantitative RT-PCR analysis of candidates. **A**. Parental MCF-7 cells. **B**. MCF-7-derived sphere cells. **C**. Quantitative RT-PCR results of six candidates (IL12RB2, CXCL1, CXCR4, ACADM, BCKDHB and HMGCS1). M indicates parental MCF-7 cells, and S indicates MCF-7-derived sphere cells.

Fig 5A and 5B show the morphologic appearance of parental MCF-7 and MCF-7-derived sphere cells. To confirm the expression levels of the candidate genes, we performed quantitative RT-PCR to determine the mRNA levels of the candidates (IL12RB2, CXCL1, CXCR4, ACADM, BCKDHB and HMGCS1) in MCF-7 and MCF-7-derived sphere cells and also measured the expression levels of SNAI and ACTIN as reference genes. The up-regulation of SNAI is associated with EMT, which is a characteristics of CSCs, and ACTIN was used as a control gene [41, 48, 49]. Quantitative RT-PCR indicated that increased mRNA expression levels for CXCL1, CXCR4 and HMGCS1 were detected in MCF-7-derived sphere cells compared with parental MCF-7 cells (Fig 5C). However, IL12RB2, ACADM and BCKDHB had no significant expression in MCF-7-derived sphere cells compared with parental MCF-7 cells (Fig 5C).

## Discussion

Meta-analysis have been widely used among scientists due to its ability to increase statistical power and provide reliable and general results in inexpensive ways and several studies have proposed meta-analysis techniques in the context of microarrays [5]. However, there is no comprehensive framework for conducting meta-analysis of microarrays [5].

In this study, using the gene set and network analysis, we proposed novel meta-analysis that integrated different gene expression profiles from several studies of tumor stem-like breast cancer cells and selected possible markers using significance and connectivity. For the significance, gene set analysis was used to select cytokine-cytokine receptor interaction, valine, leucine and isoleucine degradation, systemic lupus erythematosus and DNA replication as four significant gene sets. Among the genes of four significant gene sets, IL12RB2, CXCL1, CXCR4, ACADM, BCKDHB and HMGCS1 were selected as genes that revealed significance and up-regulation in both Affymetrix and Illumina platforms. Using the gene set analysis, our meta-analysis provided possibilities in selecting each of the individual markers considering not only statistical processes but also biological mechanisms. Because all the candidates we selected were involved in a specific pathway, our candidates offered a robust approach for explaining the mechanisms of tumor stem-like breast cancer cells.

To consider the connectivity, we conducted WGCNA and obtained the connectivity of genes in four selected gene sets. In the cytokine-cytokine receptor interaction gene set, several genes including CXCR4, CXCL1 and CXCL10 showed high connectivity in the Illumina dataset. In the valine, leucine and isoleucine degradation gene set, HMGCS1 showed high connectivity in the Affymetrix and Illumina datasets. Taken together, we selected CXCR4, CXCL1 and HMGCS1 as candidates that showed both high significance and connectivity. By adding the information of network properties, our method could suggest additional criterion to select possible biomarkers in meta-analysis.

For further validation of the expression profiles of candidate genes, we used quantitative RT-PCR and found that the mRNA expression profiles of CXCL1, CXCR4 and HMGCS1 were significantly higher in MCF-7-derived sphere cells compared with parental MCF-7 cells. Among these candidates, the chemokine receptor CXCR4 has been well documented as a mediator of metastasis in breast cancer and CXCR4-overexpressing subpopulation of cancer stem cells was reported to be essential for tumor metastasis [50–53]. Additionally, CXCL1, a proangiogenic CXC-type chemokine, is present in many cancer types, including breast, lung, pancreatic, colorectal and prostate cancers and several studies reported that CXCL1 had been identified as being overexpressed by breast cancer cells with an elevated potential to metastasize to the lung [54–58]. Because our two of the three candidates have already been confirmed as significant by several studies, our meta-analysis could provide useful approach to detect possible markers that involved in tumor stem-like breast cancer cells.

Unfortunately, there were small amount of available open datasets related to tumor stem-like breast cancer cells and only three different Affymetrix datasets from open sources were used. Also, the origins of samples were not same and the quantitative RT-PCR showed low sensitivity and robustness. With regard to breast cancer molecular subtype, except clinical samples of GSE32526, we used GSE24460, GSE35603 and E-MTAB-3860 which are expression profiles of estrogen receptor-positive lumial MCF-7 cell lines. For validation, we conducted RT-PCR by using MCF-7 cell lines which were estrogen receptor-positive luminal A subtypes. MCF-7 cell lines have been widely used to investigate the properties of cancer stem cells [59–62]. Chen et al. [59] reported high-level expression of CSC-associated properties of MCF-7 cells cultured in three-dimensional (3D) was further confirmed by high-tumorigenicity *in vivo*. Other studies also compared a luminal subtype cell line MCF-7 and mammosphere to evaluate tumor-initiating capability [61, 62].

For the data collection, we generated our gene expression profiles of Illumina platform and our gene expression profile demonstrated higher expression and connectivity than the Affymetrix datasets for the cytokine-cytokine receptor interaction and valine, leucine and isoleucine degradation gene sets. In other words, that our datasets had more distinct expression patterns in selecting classifiers than those of Affymetrix datasets. Also, by using Affymetrix and Illumina datasets, we could consider the effects derived from different platforms.

In conclusion, we demonstrate novel framework of meta-analysis that combines gene set and network analysis. Distinct from other meta-analysis, we applied the concepts of gene set analysis to our meta-analysis and considered connectivity as an additional criterion in selecting possible markers. By using the both information of significance and connectivity, we selected CXCR4, CXCL1 and HMGCS1 and, which were validated by RT-PCR. Even though, Horvath S & Dong J [19] have noted that hub genes may not always be biologically significant, we suggest that connectivity may be additional consideration for selecting candidate genes by combining gene set analysis.

## Supporting Information

S1 PRISMA Checklist (DOCX)

S1 Table Primers of gene candidates (IL12RB2, CXCL1, CXCR4, ACADM, BCKDHB and HMGCS1) used for PCR amplification. (XLSX)

S2 Table GAGE statistics from the “gage” R package of the gene sets, which were obtained from the Affymetrix and Illumina datasets. (XLSX)

S1 Fig Clustering in which m and s indicate adherent and sphere cell samples, respectively, from the GSE35603 dataset, M and S indicate adherent and sphere cell samples, respectively, from the GSE24460 dataset, and mm and ss indicate adherent and sphere cell samples, respectively, from the GSE32526 dataset. **A**. Clustering of the 15 samples, which were influenced by three different datasets **B**. After using the ComBat method, the output demonstrated that the batch effects of the different datasets were removed.
(TIF)

S2 Fig PCA plot in which M and B indicate adherent and sphere cell samples, respectively, in the Illumina dataset. Four samples from the Illumina dataset were distributed by the expression of four significant gene sets including **A**. Cytokine-cytokine receptor interaction **B**. Valine, leucine and isoleucine degradation **C**. Systemic lupus erythematosus and **D**. DNA replication.
(TIF)

S3 Fig ROC curve of four significant gene sets in Affymetrix datasets. The values of True-positive and False-positive rate were calculated from K-OPLS.
(TIF)

S4 Fig The top shows gene plots for the valine, leucine and isoleucine degradation gene set obtained from Globaltest. The red and green bars indicate genes that are up-regulated and down-regulated, respectively, in sphere cells. The bottom demonstrates that KEGG pathways include the fold-change of individual genes in the valine, leucine and isoleucine degradation gene set. **A**. Affymetrix datasets. **B**. Illumina datasets.
(TIF)
