# Supplementary figures and images for: Meta-Analysis of Tumor Stem-Like Breast Cancer Cells Using Gene Set and Network Analysis
Source: PLoS One. 2016 Feb 12;11(2):e0148818. doi: 10.1371/journal.pone.0148818 (PMC4752453; doi:10.1371/journal.pone.0148818)

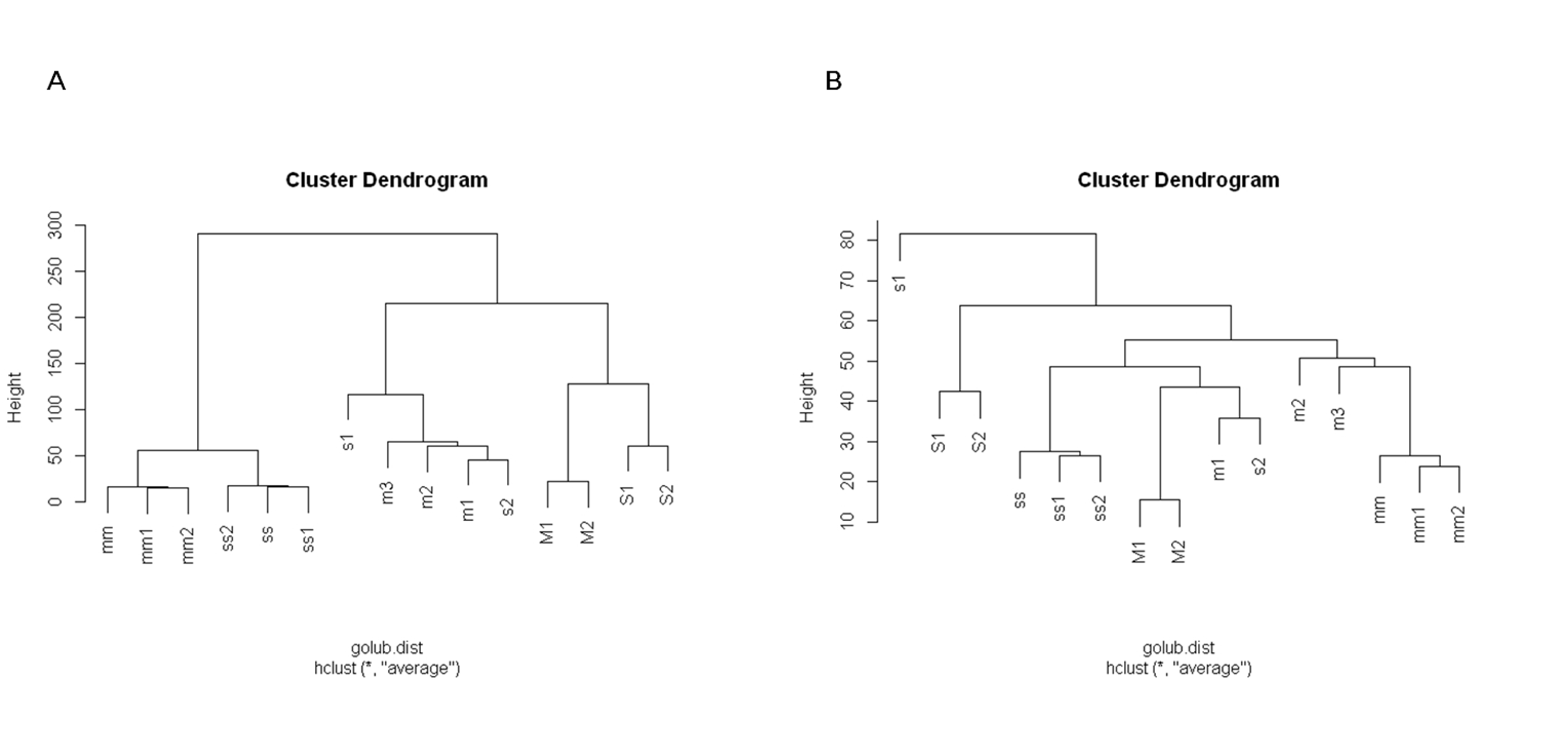

Supplement: S1 Fig — A. Clustering of the 15 samples, which were influenced by three different datasets B. After using the ComBat method, the output demonstrated that the batch effects of the different datasets were removed. (TIF) [file pone.0148818.s004.tif]

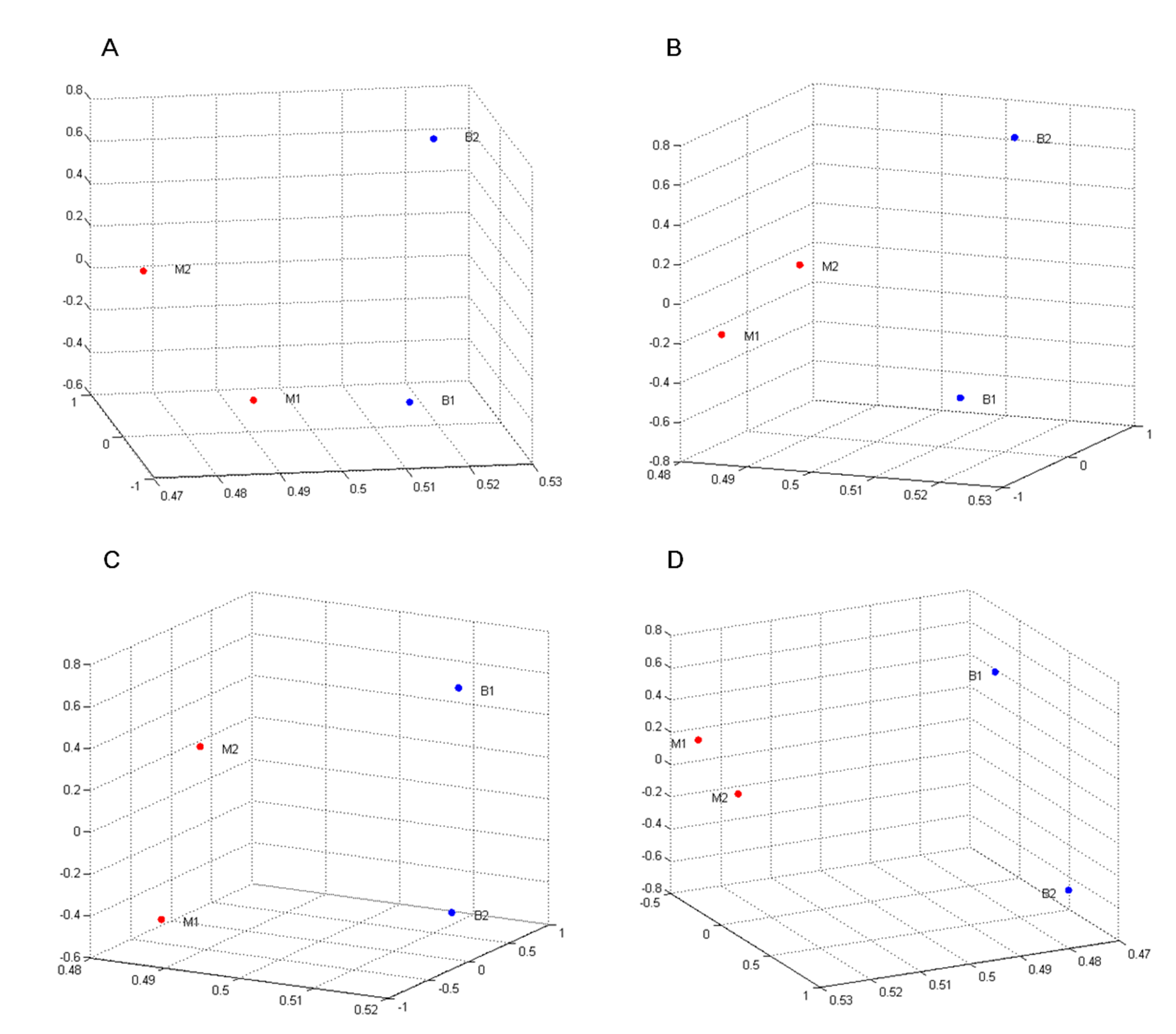

Supplement: S2 Fig — Four samples from the Illumina dataset were distributed by the expression of four significant gene sets including A. Cytokine-cytokine receptor interaction B. Valine, leucine and isoleucine degradation C. Systemic lupus erythematosus and D. DNA replication. (TIF) [file pone.0148818.s005.tif]

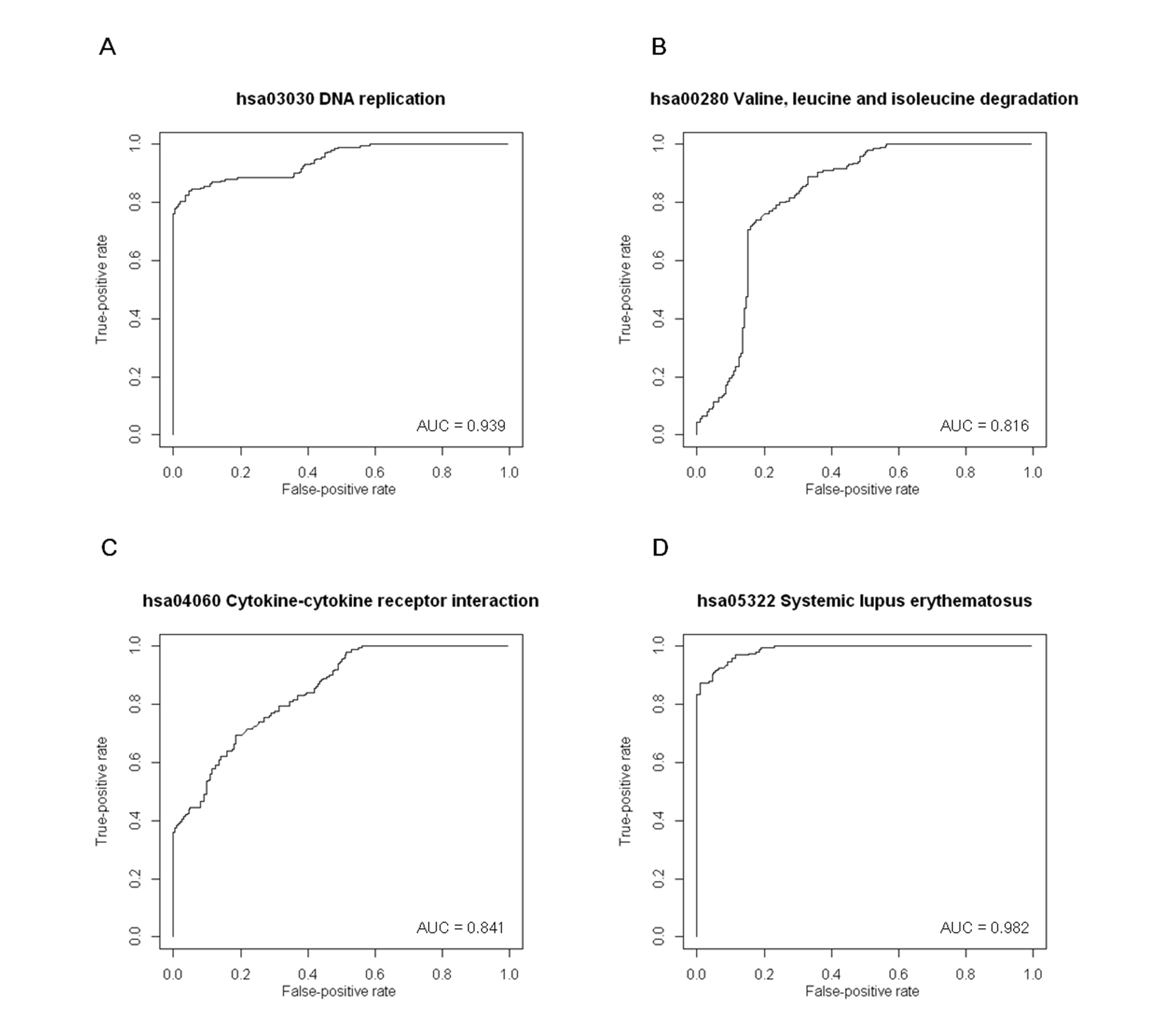

Supplement: S3 Fig — The values of True-positive and False-positive rate were calculated from K-OPLS. (TIF) [file pone.0148818.s006.tif]

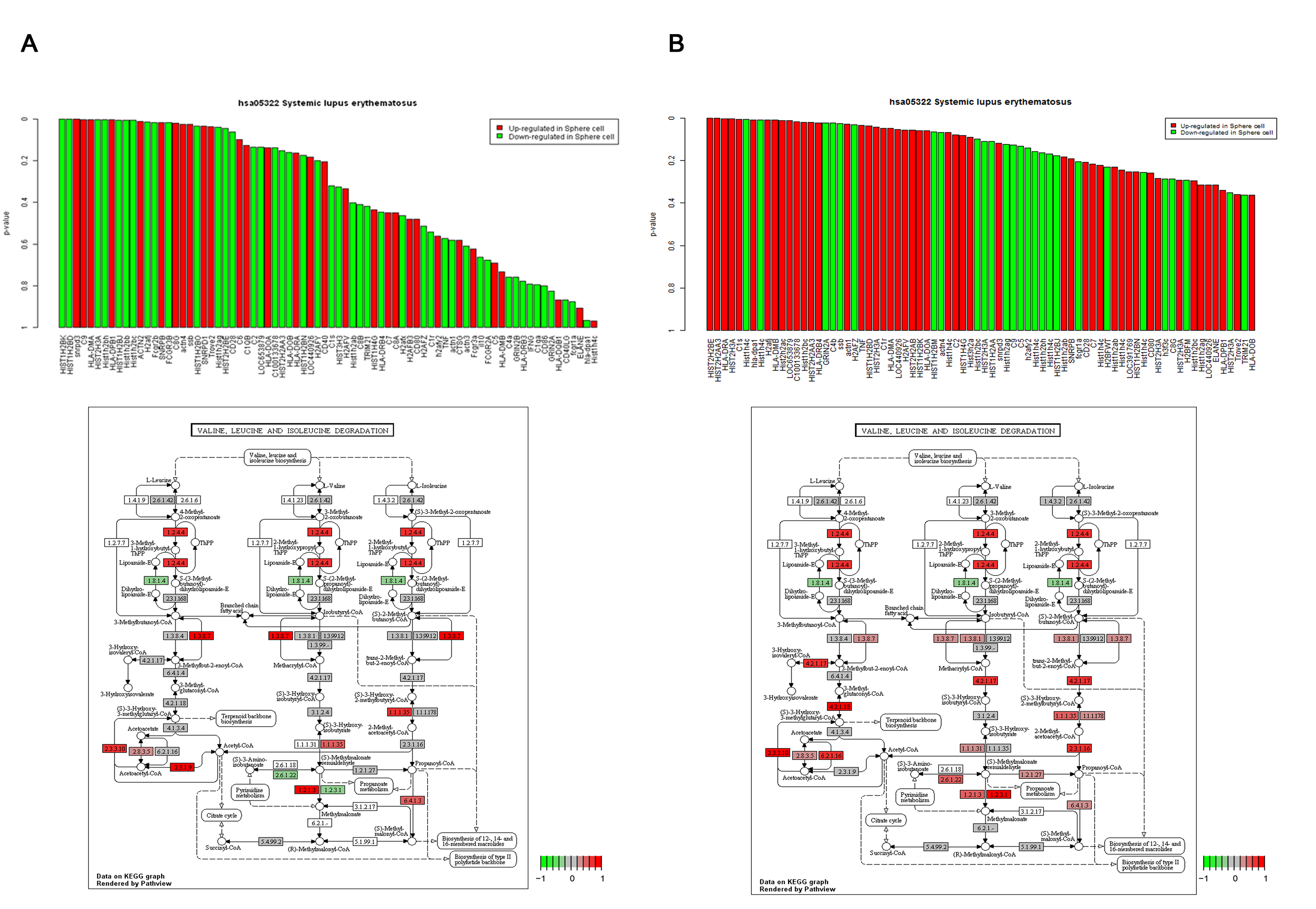

Supplement: S4 Fig — The red and green bars indicate genes that are up-regulated and down-regulated, respectively, in sphere cells. The bottom demonstrates that KEGG pathways include the fold-change of individual genes in the valine, leucine and isoleucine degradation gene set. A. Affymetrix datasets. B. Illumina datasets. (TIF) [file pone.0148818.s007.tif]
